# Potent *in vivo* efficacy of oral gallium maltolate in treatment-resistant glioblastoma

**DOI:** 10.3389/fonc.2023.1278157

**Published:** 2024-01-15

**Authors:** Mona M. Al-Gizawiy, Robert T. Wujek, Hisham S. Alhajala, Jonathan M. Cobb, Melissa A. Prah, Ninh B. Doan, Jennifer M. Connelly, Christopher R. Chitambar, Kathleen M. Schmainda

**Affiliations:** ^1^Department of Biophysics, Medical College of Wisconsin, Milwaukee, WI, United States; ^2^Department of Biomedical Engineering, Medical College of Wisconsin, Milwaukee, WI, United States; ^3^Department of Medicine, Medical College of Wisconsin, Milwaukee, WI, United States; ^4^Department of Neurosurgery, Medical College of Wisconsin, Milwaukee, WI, United States; ^5^Department of Neurology, Medical College of Wisconsin, Milwaukee, WI, United States; ^6^Department of Radiology, Medical College of Wisconsin, Milwaukee, WI, United States

**Keywords:** glioblastoma, iron, gallium, treatment, xenograft, MRI, CBV, angiogenesis

## Abstract

**Background:**

Treatment-resistant glioblastoma (trGBM) is an aggressive brain tumor with a dismal prognosis, underscoring the need for better treatment options. Emerging data indicate that trGBM iron metabolism is an attractive therapeutic target. The novel iron mimetic, gallium maltolate (GaM), inhibits mitochondrial function via iron-dependent and -independent pathways.

**Methods:**

*In vitro* irradiated adult GBM U-87 MG cells were tested for cell viability and allowed to reach confluence prior to stereotactic implantation into the right striatum of male and female athymic rats. Advanced MRI at 9.4T was carried out weekly starting two weeks after implantation. Daily *oral* GaM (50mg/kg) or vehicle were provided on tumor confirmation. Longitudinal MRI parameters were processed for enhancing tumor ROIs in OsiriX 8.5.1 (lite) with Imaging Biometrics Software (Imaging Biometrics LLC). Statistical analyses included Cox proportional hazards regression models, Kaplan-Meier survival plots, linear mixed model comparisons, and t-statistic for slopes comparison as indicator of tumor growth rate.

**Results:**

In this study we demonstrate non-invasively, using longitudinal MRI surveillance, the potent antineoplastic effects of GaM in a novel rat xenograft model of trGBM, as evidenced by extended suppression of tumor growth (23.56 mm^3^/week untreated, 5.76 mm^3^/week treated, *P* < 0.001), a blunting of tumor perfusion, and a significant survival benefit (median overall survival: 30 days untreated, 56 days treated; *P* < 0.001). The therapeutic effect was confirmed histologically by the presence of abundant cytotoxic cellular swelling, a significant reduction in proliferation markers (*P* < 0.01), and vessel normalization characterized by prominent vessel pruning, loss of branching, and uniformity of vessel lumina. Xenograft tumors in the treatment group were further characterized by an absence of an invasive edge and a significant reduction in both, MIB-1% and mitotic index (*P* < 0.01 each). Transferrin receptor and ferroportin expression in GaM-treated tumors illustrated cellular iron deprivation. Additionally, treatment with GaM decreased the expression of pro-angiogenic markers (von Willebrand Factor and VEGF) and increased the expression of anti-angiogenic markers, such as Angiopoietin-2.

**Conclusion:**

Monotherapy with the iron-mimetic GaM profoundly inhibits trGBM growth and significantly extends disease-specific survival *in vivo*.

## Introduction

Glioblastoma (GBM) is an extremely aggressive cancer for which there are limited treatment options. Despite recent advances in therapy, the prognosis remains dismal and patient survival is short (1–3). After standard therapy, which includes surgical resection followed by chemotherapy and radiation, the residual or recurrent tumor frequently becomes resistant to therapy, rendering it an extremely difficult disease to treat (4). One factor responsible for the poor response to treatment is the increase in heterogeneity of tumor cell populations comprised of predominantly treatment-resistant GBM (trGBM) cells with cancer stem cell characteristics (4, 5). This shift in genomic makeup of trGBM results in a decidedly aggressive tumor phenotype with no known cure.

Iron plays a vital role in the pathobiology of many cancers, including brain tumors, where it drives several iron-dependent processes involved in upregulated DNA repair, drug resistance, and enhanced malignant cell proliferation (6, 7). Glioblastoma iron homeostasis and iron-dependent proteins thus present attractive targets for therapeutic intervention. Extensive research has been conducted into gallium and how it interacts with tumor iron metabolism [reviewed in ref (8)]. Recently, we showed that the iron mimetic, gallium maltolate (GaM), indeed inhibits the proliferation of human GBM cell lines and GBM stem cells *in vitro* and exerts its antineoplastic effects by blocking mitochondrial function and inhibiting the iron-dependent activity of ribonucleotide reductase (9). We demonstrated that iron binds to its transport protein transferrin (Tf) and is taken up by glioma cells via transferrin receptors (TfRs) that populate the cell surface. Moreover, early *in vivo* data suggest a disruption of tumor iron homeostasis indicated by a retardation of tumor growth in an orthotopic rat brain tumor xenograft model following a continuous intravenous infusion of GaM for ten days.

We have since developed a stable radioresistant glioma model involving *in vitro* irradiated GBM cells (10). We demonstrated that *in vitro* irradiation causes the cells to undergo an epithelial to mesenchymal transition, a process related to gaining stem cell properties and subsequently, resistance to treatment. Initial *in vivo* evaluation of these irradiated cell lines produced a clinically-relevant intracranial xenograft model mimicking MRI and histopathological findings seen in patients with trGBM (11, 12). Consequently, we hypothesized that targeting the diverse gallium-sensitive GBM cell populations via their inherent iron metabolism would be an intriguing approach to combat trGBM. The goal of this study is to investigate the antineoplastic effects of oral GaM in a rat xenograft model of experimentally-induced trGBM.

## Materials and methods

### Cell culture

All media and supplements were purchased from Life Technologies™ (Grand Island, NY, USA), unless stated otherwise. As previously described, adult U-87 MG glioblastoma cells (HTB_14™; ATCC^®^, Manassas, VA, USA) were irradiated with five doses of 2.09 Gy/min (total radiation dose of 10 Gy) to yield the treatment-resistant U87-10Gy cells used in this study (13). Irradiation of brain tumor cells results in upregulation of genes promoting tumor aggressiveness and invasion, conferring radioresistance (10). The cells were cultured in MEM with Earle’s salts fortified with 10% FBS, supplemented with 1% sodium pyruvate and 0.1% Gentamicin, and maintained at 37°C in a humidified atmosphere of 5% CO_2_. All media and supplements were sourced from Gibco™, ThermoFisher Scientific, Waltham, MA, USA.

### Animals

Care of the animals before and during the experimental procedures was conducted in accordance with the policies of the NIH Guide for the Care and Use of Laboratory Animals. All protocols were approved by the Institutional Animal Care and Use Committee at the Medical College of Wisconsin. Methods are provided in accordance with ARRIVE guidelines. For this pilot study, 18 male and 20 female athymic rats weighing approximately 180-250g were obtained from Envigo RMS, Inc. (Indianapolis, IN, USA) and housed in pairs within individually ventilated cages. Female rats were included to avoid gender bias and account for potential gender differences in response to various chemotherapeutics (14–16). Preliminary sample size (n = 5 per group) was determined previously by power analysis using parameters to reflect an expected response in 75% of the animals, α = 0.05, β = 0.2, power = 0.8. Animals received an irradiated laboratory diet and RO purified water *ad libitum*.

### Tumor cell inoculation

We slightly modified our previously published xenograft model protocol to include inhalant anesthesia to ensure a steadier plane of anesthetic depth during the procedure, as well as allow for a smoother induction and recovery (9). Briefly, rats were anesthetized with isoflurane (Phoenix Pharmaceutical, Inc., St. Joseph, MO, USA) delivered in medical grade oxygen. Once appropriate anesthetic depth was ascertained by lack of response to toe pinch, animals were placed on a warm surface in a stereotaxic device with non-penetrating ear bars (Stoelting Co., Wood Dale, IL, USA) to keep the head immobilized. After standard aseptic surgical preparation, a 1-inch midline incision was made in the skin on the top of the head. Using a sterile burr, a 0.9 mm hole was drilled into the skull 2 mm lateral and 1 mm anterior of the bregma to facilitate the implantation of 200,000 U87-10Gy cells into the right frontal lobe at a depth of 3 mm relative to the dural surface. The injection occurred over five minutes at a steady rate of 2 μL/min (Harvard NANOmite syringe pump, Harvard Apparatus, Holliston, MA, USA), after which the needle was left stationary for five more minutes and then slowly withdrawn manually over an additional five minutes. Finally, the skin was closed with Vetbond™ tissue adhesive (3M, St. Paul, MN, USA), and the animals were allowed to recover in clean, prewarmed cages. Animals were monitored daily following surgery and throughout the study period. Health status and body condition were assessed as described previously (17).

### Gallium maltolate treatment

In the clinical setting, chemotherapy, such as temozolomide, typically is administered orally (18). However, preclinical rodent models frequently rely on the intravenous or intraperitoneal injection of study drugs, which does not practically reflect the clinical situation. To more accurately parallel the clinical experience in which patients with trGBM may be treated daily with oral chemotherapy, we administered GaM (Gallixa, LLC, Menlo Park, CA, USA) in an oral preparation. On day seven post-implantation, rats began training to voluntarily ingest commercial hazelnut spread (Nutella^®^, Ferrero U.S.A., Inc., Somerset, NJ), which would serve as drug vehicle (19). For the first three days, cage mates received 1.0g Nutella^®^ together. On the remaining two days, rats were fed 0.50 to 1.00g Nutella^®^ individually, to gauge individual appetites. Ultimately, approximately 0.40 – 0.50g was judged to be the appropriate amount that could be easily and completely ingested in under 30 seconds by most animals.

Tumor take was confirmed on day 14 post-implantation by MRI, as described below. Animals then received oral GaM at a dose of 50mg/kg/day. Each animal was weighed daily to calculate the appropriate amount of GaM powder to be mixed into a fresh aliquot of Nutella^®^ and to monitor the animal’s health. Each dish containing a GaM preparation was weighed before and after ingestion to determine the amount of GaM ingested. To mimic the clinical patient experience of receiving cycles of chemotherapy, rats were put on a two-week on, one-week off treatment cycle with treatment effect gauged by weekly MRI surveillance.

At the time of necropsy, urine samples were collected when possible. Analysis for basic hepatic and renal function was conducted using commercially available test strips (HEALTH MATE™ VET-10 and One+Step^®^ Vet Kidney Disease Tests, DFI Co. Ltd., Republic of Korea).

### MRI data acquisition

A prominent feature of U-87 MG xenograft tumors is their profoundly leaky vasculature, consisting of inefficient and dysfunctional vessels, unable to perfuse the tumor tissue effectively (20–22). This renders this model especially attractive for MR imaging studies, since it produces strong enhancement after contrast agent administration (23, 24). In our experience, this feature and the expansive tumor size allows for collection of quality advanced MRI data (12, 25, 26). Thus, for translatable development of imaging biomarkers, orthotopic xenograft tumors were the most suitable choice for our studies. *In vivo* advanced MR imaging was carried out weekly starting on day 14 post-implantation of tumor cells into the brain. All preclinical MR imaging was performed on a 9.4T Bruker BioSpec 94/20 USR preclinical scanner. Images (FOV=3.5 cm) were acquired as part of our custom-tailored preclinical acquisition protocol. All examinations included pre- and post-contrast T1-weighted (T1W) anatomical images (TR/TE=1500/minimum, matrix=256x256), pre- and post-contrast T2W GRE scans [TR/TE=4500/(2:2:24), matrix=128x128], and dynamic susceptibility contrast imaging acquired using a T2*W EPI sequence (DSC; TR/TE=1000/40, 120 repetitions, matrix=96x128). A bolus of 5 mg/kg magnetic iron oxide nanoparticles (Molday ION, BioPAL Inc., Worcester, MA, USA) was injected halfway into the DSC scan at a rate of 10 ml/min using a power-injector. Post-contrast T2W GRE scans were acquired following the DSC, and post-contrast T1W scans were acquired following the additional injection of 0.2 mmol/kg gadobutrol (Gadavist^®^: Bayer HealthCare Pharmaceuticals Inc., Whippany, NJ, USA).

### Image & data analysis

Acquired MRI data was processed using Imaging Biometrics’ analysis software (Imaging Biometrics LLC, Elm Grove, WI) and custom-made MATLAB scripts. To determine enhancing tumor free of confounding pre-contrast bright signal, quantitative delta-T1 maps (dT1) were generated from calibrated pre- and post-contrast T1W images using the IB Delta Suite™ plugin in Horos 8.5.1 (Lite) (Pixmeo SARL, Geneva, Switzerland) (27). R2* maps were generated from pre- and post-contrast T2*W images, which were then used to generate standardized steady-state cerebral blood volume maps (ssCBV). Tumor volume (mm^3^) was extracted from regions of interest (ROIs) drawn manually on dT1 maps in Horos. The same ROIs were imported into ssCBV maps to extract mean tumor ssRCBV values (a.u.). Standardized relative cerebral blood volume maps (rCBV) were generated from DSC scans (26).

### Statistical analyses

Kaplan-Meier survival curves (Gehan-Breslow analysis) were constructed in SigmaPlot 12.5. (Systat Software, Inc., San Jose, CA, USA). Student’s t-test was applied for group comparisons at individual timepoints. Cox proportional hazards regression models were used to probe the relationship between treatment and disease-specific overall survival (OS). Because it has been suggested that tumor site may influence outcome (28, 29), we also wanted to determine whether this was a confounder. Since all tumors were injected into the right striatum, we restricted our analysis to whether day 14 baseline tumors established superior or inferior to the corpus callosum. Because postoperative, residual enhancing tumor volume has been correlated with outcome in GBM (30), we set out to see whether this was a confounder in our study or not. Hence, covariates utilized for multivariable Cox regression analyses included sex, tumor volume at baseline, as well as location relative to the corpus callosum at baseline. These features were fed into a multivariable Cox Hazard regression model stratified by treatment group. Wald Chi-Square statistic was used to rank the contribution of these variables to the model. T-statistic for slopes comparison was taken as an indicator of tumor growth rate. Longitudinal group comparisons were made using Linear Mixed Model (LMM) analysis in IBM SPSS Statistics 26. Akaike’s Information Criterion (AIC) was used to test the fitness of the random intercept model with and without introduction of random slopes. The preferred model for LMM analysis was determined to be the one with the lower AIC for the dependent variables. Significance level was set at *P* = 0.05.

### Immunohistochemistry

Brains were obtained for histological analysis from rats exhibiting signs of morbidity. Following humane euthanasia, the removed brains were fixed in 10% buffered formalin solution. Standard processing, sectioning, and staining for all tissues were carried out by the Children’s Research Institute Histology Core at Children’s Wisconsin. All immunohistochemical (IHC) staining was performed on a Leica Bond Rx automated staining platform (Leica Microsystems GmbH, Wetzlar, Germany). Briefly, 4 µm brain sections underwent deparaffinization, serial rehydration, and chemical antigen retrieval before peroxidase, protein, and biotin blocking (DAKO Peroxidase Block, S-200389-2, Agilent Technologies, Santa Clara, CA, USA; Biocare Background Sniper, BS966L, Biocare Medical, Pacheco, CA, USA; Avidin/Biotin Blocking System, SP-2001; Vector Laboratories Inc., Burlingame, CA, USA). The sections were then incubated with primary antibodies for 30 to 60 minutes at room temperature: cellular proliferation (1:50 KI67 DAKO monoclonal mouse anti-rat, clone MIB-1, M7240; Agilent Technologies, Santa Clara, CA, USA), vascular endothelium (1:1000 von Willebrand Factor DAKO polyclonal rabbit anti-human, A0082; Agilent Technologies, Santa Clara, CA, USA), transferrin receptor 1 (1:1000 CD71, polyclonal rabbit anti-rat, #254553, ABBIOTEC™; San Diego, CA, USA), Ferroportin (1:400, polyclonal rabbit anti-rat, PA5-22993, ThermoFisher Scientific, Waltham, MA, USA), hypoxia (1:300 Hif-1a monoclonal mouse anti-human, clone ESEE122, ab8366, Abcam, Waltham, MA, USA), and angiogenic factors (1:100 VEGF monoclonal mouse anti-human, C-1, sc-7269, Santa Cruz, Dallas, TX, USA; 1:1000 ANG2 polyclonal rabbit antibody, ThermoFisher Scientific, Waltham, MA, USA). Following application of the appropriate biotinylated secondary antibody for 30 minutes, antigen detection was performed using a streptavidin-HRP immunoperoxidase (Vector labs Streptavidin HRP SA-5004-1, Vector Laboratories, Inc. Newark, CA, USA) technique. For visualization, diaminobenzidine (DAB+, DAKO K3467; Agilent Technologies, Santa Clara, CA, USA) was applied for 3-5 minutes. Finally, brain tissues were counterstained with hematoxylin before undergoing serial dehydration, clearing and coverslipping with synthetic mounting media.

### Immunohistochemical analysis

Visual analysis of IHC staining was performed with a Nikon^®^ Eclipse 80i microscope equipped with a MicroPublisher 3.3 RTV color video camera (Q Imaging, Surrey, BC, Canada). Images were captured using NIS elements imaging software (Version 7.0, Nikon Instruments, Inc., Melville, NY), and analyzed using ImageJ 1.52c (http://imagej.nih.gov/ij). For determination of mitotic index (MIB-1%), high magnification images (400X) were taken across the largest tumor diameter of lesions stained for KI67. The percent of positive staining cells was determined out of a total of 1,000 cells counted for each tumor.

## Results

The presence of tumor could not be confirmed on day 14 in three rats (one male, two females) in the control group and 6 rats (one male, five females) in the GaM group. These animals were excluded from further analysis. **Supplementary Figure 1** shows the replicability of the tumor-suppressive effects of oral GaM in the experimental batches included in this study. Hence, pooled data for 12 control (n = six male and six female) and 16 GaM-treated (n = nine male and seven female) animals is presented, with sub-analyses by sex provided when indicated.

The overall tumor take was 88.2% in male and 65.0% in female rats, representing an incidence ratio of 1.36. Mean compliance with voluntary ingestion of GaM was 94% (47 mg/kg/day) in the treatment group, with slight fluctuations in appetite apparent for 24 to 48 hours following anesthetic events (MRI sessions in all animals) and during estrus (females). In male rats, the compliance of GaM ingestion was 92% (46 mg/kg/day) and in females 95% (48 mg/kg/day). No adverse effects to body condition or changes in behavior due to GaM consumption were noted. Weekly weight gain from days 14 to 28 in males was comparable between GaM and control groups (2.48% and 2.68%, respectively; *P* = 0.916). Similarly, there were no significant differences in weight gain from days 14 to 35 in female animals of either group (1.30% GaM-treated, 2.68% controls, *P* = 0.593).

### Oral GaM extends disease-specific survival and improves quality of life

**Figure 1:**
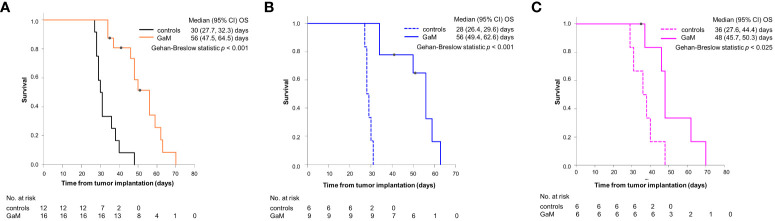
Survival analysis for GaM-treated and untreated animals. **(A)** Kaplan–Meier curves for OS pooled by treatment, **(B)** in males, and **(C)** in females. ○ = censored; one male animal was euthanized on day 50 to verify observed imaging changes implying tumor resolution, one further male and one female were pulled from the study due to unrelated development of pododermatitis.

The median OS for GaM-treated animals was 56 days (range: 34-70 days) and 30 days (range: 27-48 days) for controls (*P* < 0.001) (**Figure 1A**). Cox regression analysis confirmed a significant correlation between treatment with GaM and OS (b = 2.270, SE = 1.107, *P* = 0.0256), but neither sex, nor baseline tumor volume, nor location had a significant impact (*P* = 0.978, *P* = 0.623, and *P* = 0.799, respectively). Although sex did not appear to influence outcome, GaM-treated males did seem to have a greater survival benefit vs. untreated controls (median OS = 56 vs. 28 days, respectively; *P* < 0.001) than GaM-treated vs. untreated females (median OS = 48 vs. 36 days, respectively; *P* = 0.025) (**Figures 1B, C**).

GaM-treated animals did not exhibit any significant neurological symptoms prior to death despite demonstrable lesion expansion. The only signs of morbidity in these rats were a gradual decline in appetite with mild depression and slowing in activity 24-36 hours prior to death. Hepatic and renal function parameters determined from urine samples taken at the time of euthanasia (n = five) were all within normal limits. On necropsy, all internal organs of treated animals appeared normal and healthy without any gross lesions, discoloration, or changes in texture. Body condition at the time of death was judged to be ‘good’ to ‘very good’, based on overall body condition, the groomed state of the coat and the presence of abundant omental fat and food in the alimentary canal. Due to their naturally larger size, males had more pronounced abdominal and subcutaneous fat deposits than females.

In contrast, and despite supportive treatment, animals in the control group all exhibited a progressive worsening of body condition (rough coat and skin, sunken eyes and flanks, marked porphyria, depression, and loss of appetite) in the 48-72 hours prior to death (**Supplementary Figure 2**). Neurological deficits were evident in these animals and progressively worsened during that same time period. On necropsy, animals were judged to be in “poor” to “fair” condition, based on absence of omental fat and minimal presence of food in the alimentary canal. Intestines appeared heavily congested.

### Oral GaM inhibits tumor growth

**Figure 2:**
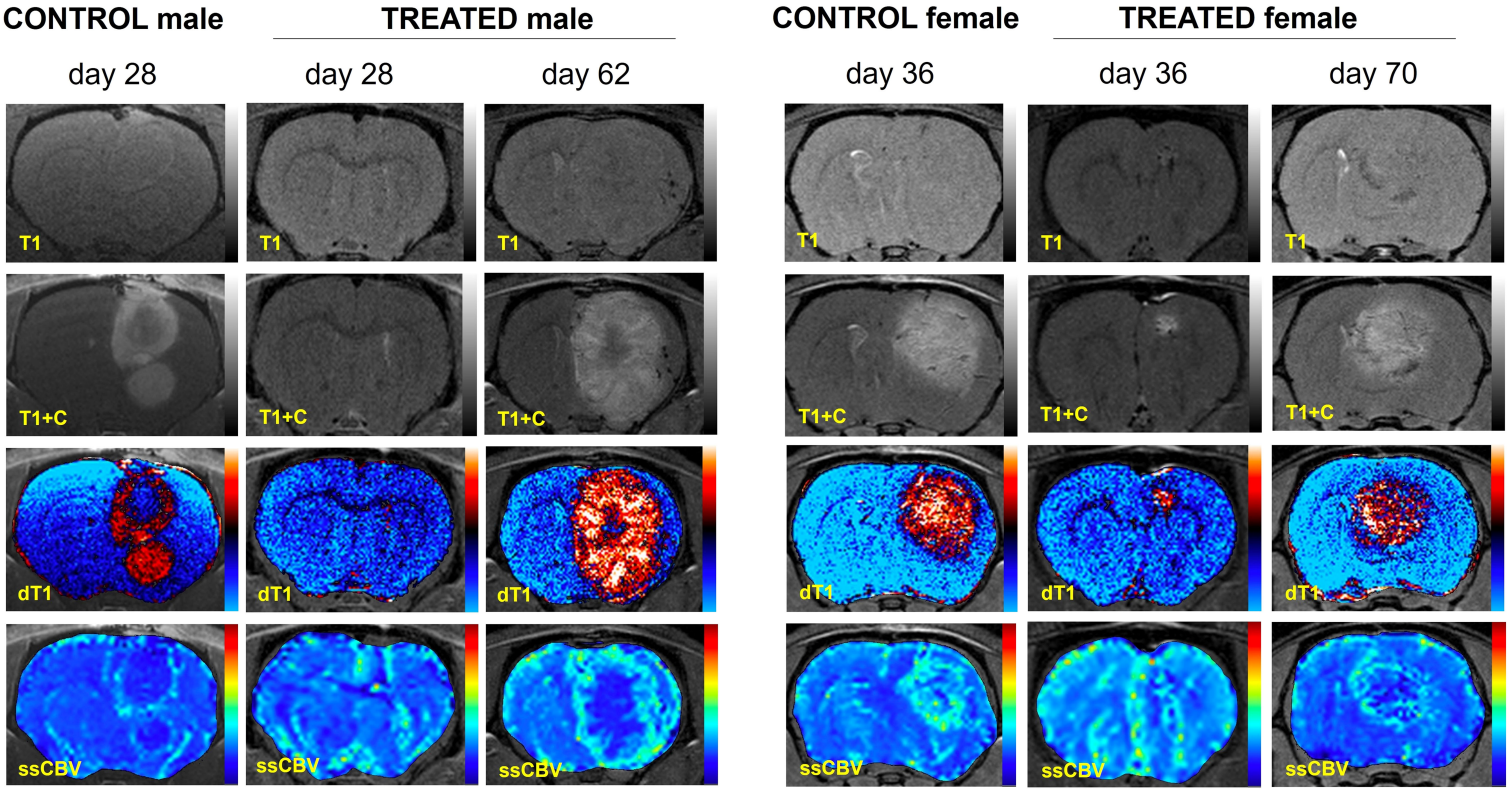
Advanced MRI characterization of tumor growth in four representative control and GaM-treated animals. Since T1 effects (first row) may confound post-contrast enhancement (T1+C, second row), quantitative dT1 maps (3^rd^ row) were constructed to show true enhancement. Perfusion imaging (ssCBV, fourth row) indicates characteristic GBM tumor vascularization patterns. Taken together, dT1 and ssCBV highlight the slow progression of tumor growth in treated animals compared to untreated controls.

**Figure 3:**
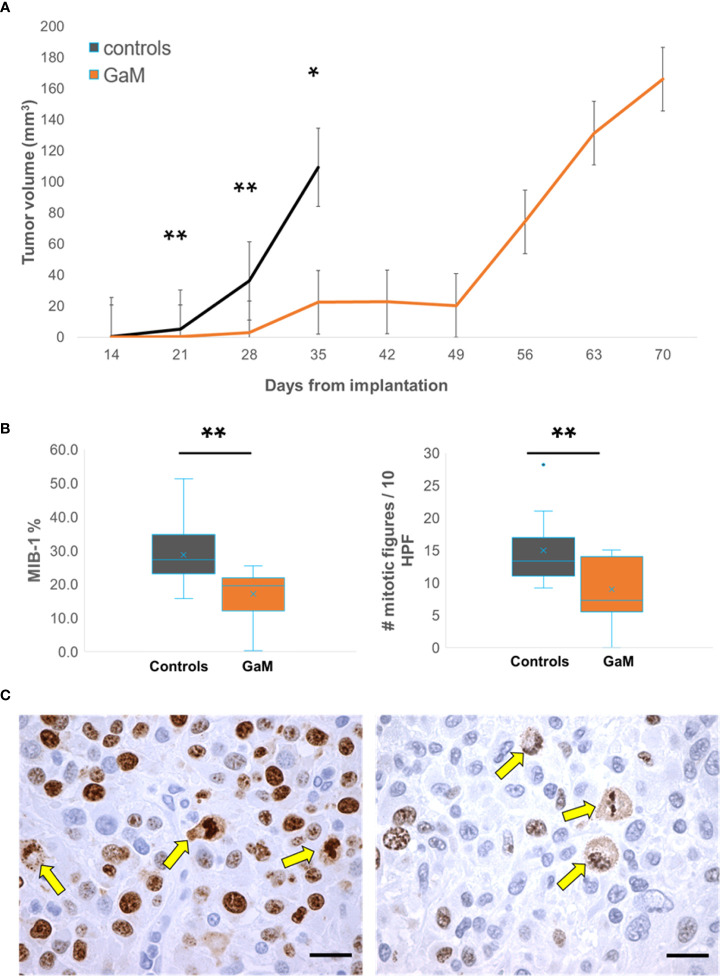
Treatment with oral GaM inhibits cellular proliferation *in vivo*. **(A)** Overall, treated trGBM tumors exhibited a significantly slower mean weekly tumor growth rate than untreated controls at matched timepoints and beyond. **(B)** Xenograft tumors were examined for proliferation markers using immunohistochemistry (brown stain). Shown are representative images from an untreated control (left) and an animal treated with GaM. Arrows indicate mitotic figures. Light microscopy, KI67; scale bar = 20 μm. **C)** Treatment with GaM resulted in a significant reduction in proliferation markers. Linear mixed model analysis and paired Student’s t-test (equal variance) * *P* < 0.05. ** *P* < 0.01.

**Figure 2** shows example MRI results characterizing the growth patterns of control and GaM-treated xenograft tumors at an early timepoint in comparison to the treated tumor at a later timepoint. Examples from both male and female rats are shown. Treated tumors develop at a significantly slower rate, taking up to twice as long to reach volumes comparable to those of controls. Additional imaging biomarkers obtained, including delta-T1 (dT1) and steady-state cerebral blood volume (ssCBV) maps (3^rd^ and 4^th^ rows), show enhancing tumor, free of confounding bright signal from blood products and increased vascularity on the tumor rim. Through day 35, GaM-treated xenograft tumors grew significantly slower than control tumors at respective rates of 4.35 mm^3^/week and 23.56 mm^3^/week, (*P* < 0.001). This suppressed growth rate was sustained until day 49 in the treatment group (5.76 mm^3^/week), after which it dramatically increased (25.29 mm^3^/week). Tumor volumes and growth rates did not differ significantly between males and females in either control or treatment groups. Pooled, and in accordance with the observed slower growth rate, tumor volumes of treated animals were significantly smaller than those in controls on days 21 (*P* = 0.0037), 28 (*P* = 0.0059) and 35 (*P* = 0.011) (**Figure 3A**). Treatment with GaM also resulted in a significantly lowered proliferation index MIB-1% (*P* = 0.0026) and mitotic figures (*P* = 0.0093), as illustrated in **Figures 3B, C**. Although this trend was observed in both sexes, it was only significant in males (**Supplementary Figure 3**).

**Figure 4:**
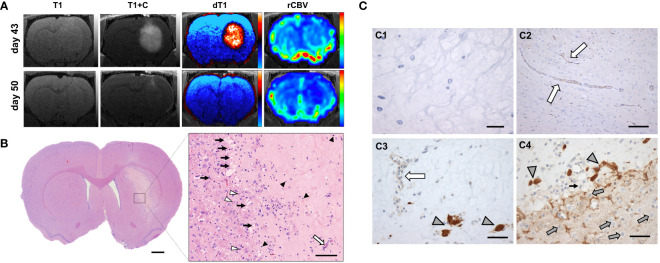
Complete resolution of trGBM tumor in one GaM-treated animal. **(A)** Advanced MRI dT1 and rCBV maps suggest a resolution of the lesion over the course of a week. **(B)** On histological examination, widespread necrosis due to treatment effect was evident. A close-up view of the central part of the lesion shows capillary ingrowth along the lesion boundary (black arrows), indicative of a reparative process. Normal healthy vessels (white arrow) are further evidence of tumor regression and tissue healing. Some lymphocytes (black arrowheads) remain, pointing to an ongoing inflammatory process in response to the presence of necrosis. A few apoptotic cells were identified (white arrowheads). **(C1)** Sparse cellularity and absence of staining for KI67 proliferation markers supports absence of viable tumor. **(C2)** Vessels within the lesion were normal in appearance. **(C3,C4)** Transferrin receptors were overexpressed in few remaining tumor cells (gray arrowheads), but not in normal vasculature (white arrow). Of note are non-tumor TfR-negative cells (gray arrows) beyond the lesion margins. Light microscopy, H&E, vWF, CD71; scale bars = 1 mm **(B)**, 100 µm (B pop-out), 20 μm **(C1)**, 100 µm **(C2)**, 50 µm **(C3, C4)**.

Of note, one GaM-treated animal exhibited a 93% reduction in contrast-enhancing lesion volume and a 69% reduction in relative cerebral blood volume (rCBV) on day 50, with all advanced MRI parameter maps indicating complete resolution of the lesion (**Figure 4A**). To determine the reason for these imaging changes, this animal was euthanized for histological analysis. Immunohistochemical findings in this animal confirm a profound treatment effect, indicated by widespread necrosis throughout the lesion and evidence of healing along its periphery (**Figure 4B** pop-out and **4C1**). In addition, no invasive margins were identified. Moreover, all vasculature and surrounding brain structures were judged to appear normal and healthy by a neuropathologist (**Figure 4C2,3**). Evidence of TfR upregulation as an indicator of iron deprivation was noted in few remaining non-viable tumor structures (**Figure 4C3,4**).

Two GaM-treated male animals experienced more than doubling of tumor volume on day 28 compared to the previous imaging time point. Therefore, the decision was made to continue oral GaM administration in these two rats, while all others were taken off treatment for seven days before resumption of therapy. However, despite continued daily treatment of the two GaM-treated animals, they exhibited neurological signs (ataxia, head tilt) on day 34, necessitating humane euthanasia. One female from the GaM group also succumbed early to rapid lesion expansion on day 37. In these animals, as for other GaM-treated animals, notable cell distension was observed in tumor tissues corresponding to central non-enhancing areas and likely necrotic areas on MR images in GaM-treated animals (**Supplementary Figure 4**). Moreover, necrosis observed in treated animals was histologically different from controls with evidence of programmed cell death (**Supplementary Figures 5A, B**). Controls exhibited signs of uncontrolled necrosis (**Supplementary Figure 5C**).

### GaM therapy prevents invasion

**Figure 5:**
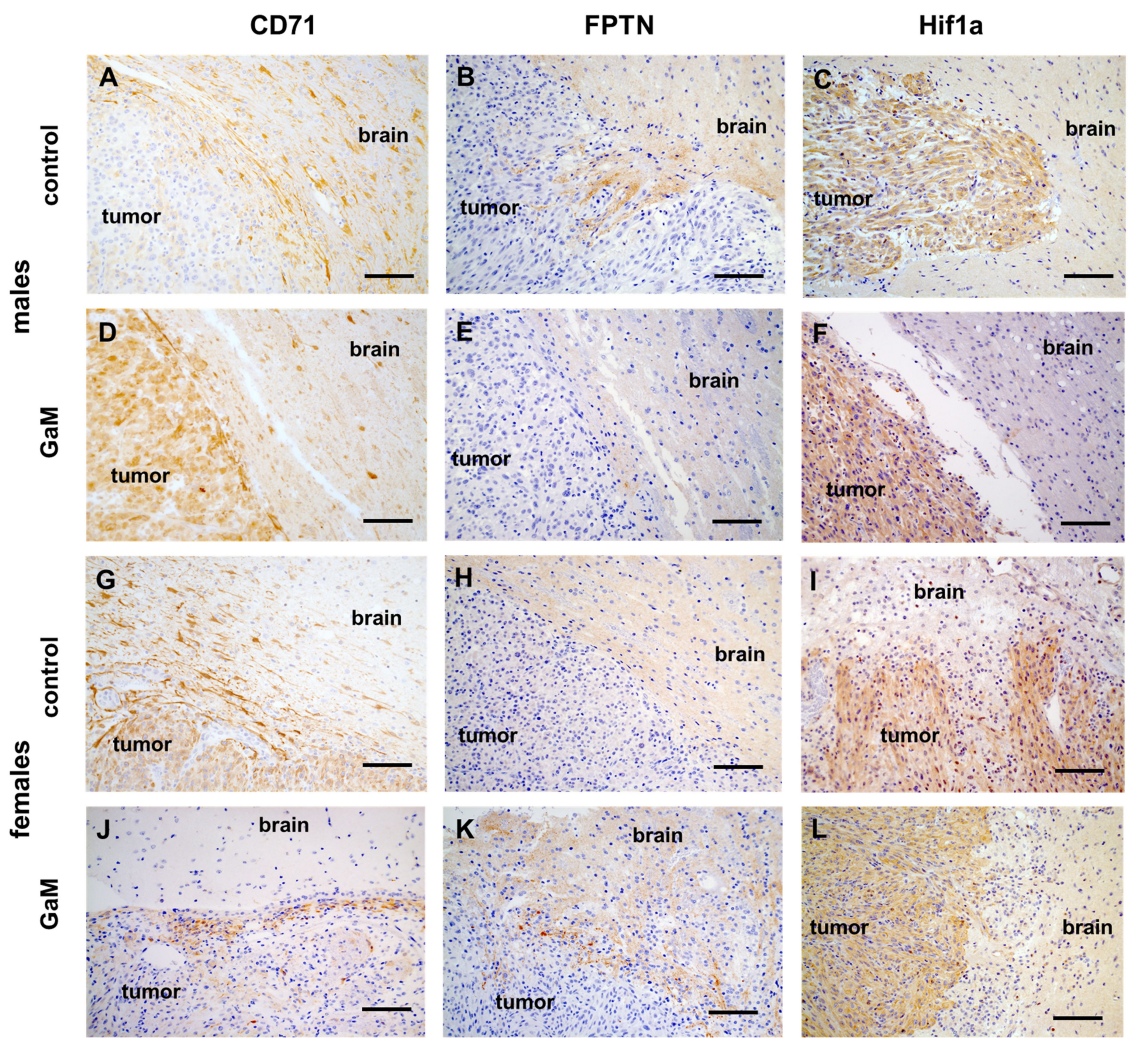
Immunohistochemical expression of markers of iron metabolism. **(A, G)** Positive TfR (CD71) staining (brown) beyond an untreated xenograft tumor is consistent with the beginning of an invasive edge. **(D, J)** Treatment with GaM prevents invasion of adjacent brain parenchyma. **(B, E, H, K)** Ferroportin (FPTN) expression was only detected in normal brain parenchyma and not within the tumor. **(C, I)** Hypoxia Hif1a was detected in tumor tissue but not surrounding brain parenchyma. **(F, L)** treatment with GaM did not change this expression pattern. Light microscopy, scale bars = 50 µm. .

Untreated xenograft tumors were characterized by an active invasive edge (**Figures 5A–C, G, I**). TfR-expressing cells were observed far beyond the tumor proper in adjacent brain tissue (**Figures 5A, G**). In treated xenograft tumors, this invasive edge was abolished with a clear demarcation between tumor and brain (**Figures 5D–F, J–L**). In response to GaM treatment, TfRs were starkly upregulated in tumor tissue, but not adjacent brain, reflective of iron deprivation (**Figures 5D, J**). Interestingly, ferroportin, expressed solely in the brain parenchyma and not in tumor tissue (**Figures 5B**, **H**), did not appear to be affected by treatment (**Figures 5E, K**). Similarly, Hif-1 alpha expression, which was abundant in tumor tissue compared to adjacent brain parenchyma, did not appear to significantly differ between untreated (**Figures 5C, I**) and treated tumors (**Figures 5F, L**).

### GaM therapy results in vascular changes

**Figure 6:**
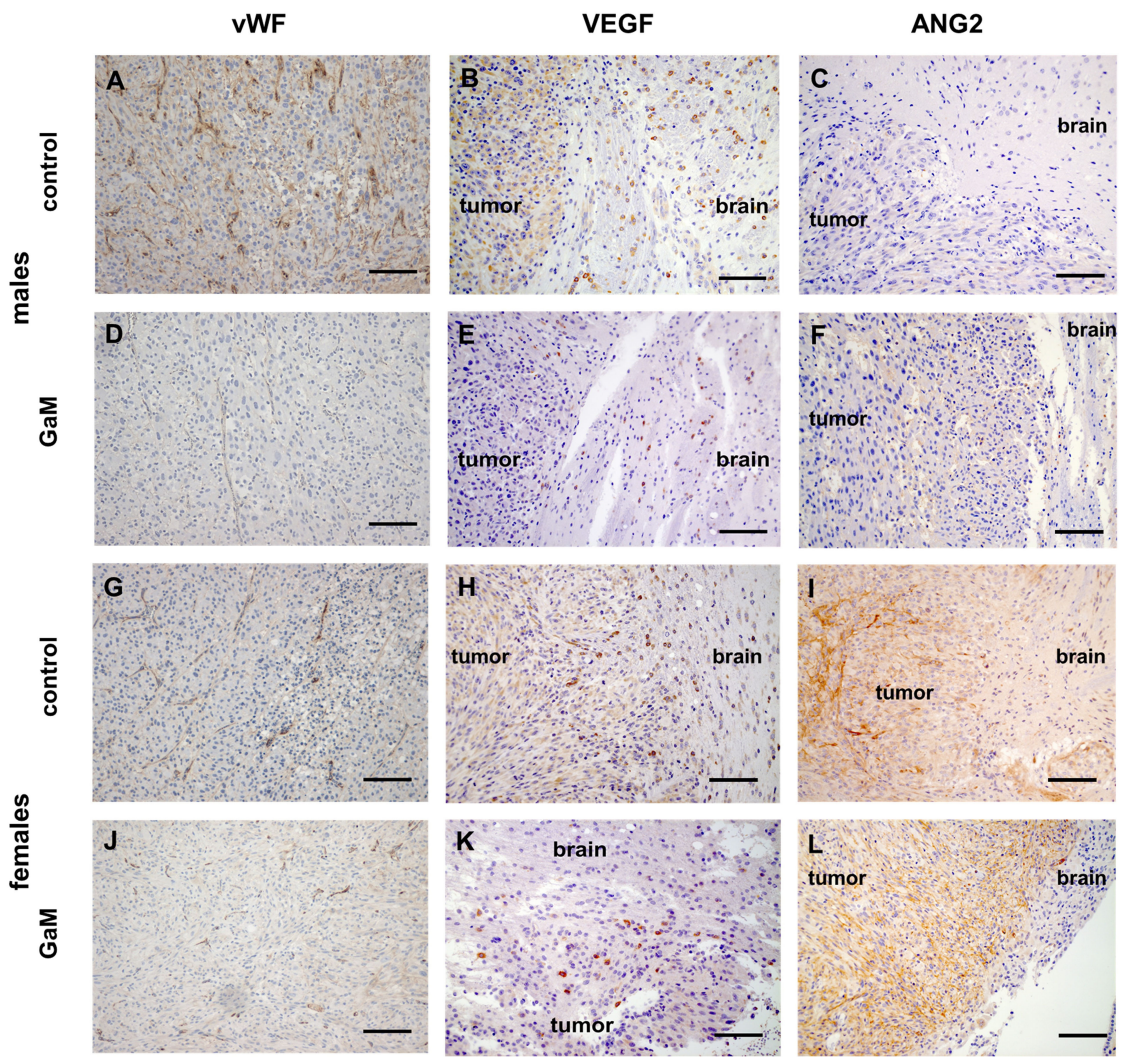
Treatment with oral GaM effects changes in angiogenic markers. Tumor vessels in untreated controls **(A, G)** readily express vWF, whereas GaM therapy decreases both vascular density and vWF expression **(D, J)**. Similarly, and unlike their untreated counterparts **(B, H)**, GaM-treated tumors express less pro-angiogenic VEGF **(E, K)**. Compared to controls **(C, I)**, anti-angiogenic ANG2 expression is upregulated following GaM therapy **(F, L)**. Light microscopy, scale bars (vWF) = 100 mm, scale bars (VEGF and ANG2) = 50 µm.

Compared to untreated controls (**Figures 6A–C, G–I**), vessels of GaM-treated tumors exhibited signs of normalization, characterized by lower vessel density, prominent vessel pruning, loss of branching, and uniformity of vessel lumens (**Figures 6D, J**). Concurrently, downregulation of VEGF was observed in treated (**Figures 6E, K**) tissues. Interestingly, tumors in female rats expressed high levels of angiopoietin 2 (ANG2) before and after treatment (**Figures 6I, L**), while in males a slight upregulation was observed only following treatment with GaM (**Figure 6F**). Tumor perfusion in the GaM group was significantly lower on day 35 than in the control group (*P* = 0.033) (**Supplementary Figure 6A**). Moreover, sex-related differences in perfusion parameters in both control and treatment groups were observed (**Supplementary Figure 6B**). Females had consistently higher perfusion values in both control and treatment groups, compared to males. On days 14, 21, and 28 post tumor implantation, the ssCBV of untreated females was significantly higher than that of untreated males (*P* = 0.0003, *P* = 0.00008, and *P* = 0.0023, respectively). In GaM-treated rats, significant differences in ssCBV between the sexes were only noted on day 28 post tumor implantation (day 14 of GaM administration) (*P =* 0.0203). A significant blunting of tumor perfusion was noted in GaM-treated females on days 21 and 28 post tumor implantation (days 7 and 14 of GaM administration) (*P* = 0.0031 and *P* = 0.0244, respectively). Upticks in ssCBV values were noted to precede increases in tumor volume in treated animals.

## Discussion

This study demonstrates the efficacy and safety of *oral* GaM for treatment-resistant glioblastoma *in vivo*. We recently demonstrated that the novel iron mimetic, GaM, when administered intravenously, has antineoplastic activity against the growth of GBM xenograft tumors *in vivo* (9). We further elucidated the mechanisms *in vitro* by which GaM interferes with GBM iron metabolism. In continuation of our earlier work, we now show for the first time that the novel approach of an *oral formulation* of GaM exhibits profound antitumor efficacy in GBM refractory to treatment.

Iron and its transport and storage proteins play a crucial role in cancer cell proliferation (31, 32). Dysregulation of the fine-tuned balance of this metabolic process by knockdown or inhibition of any of these proteins and receptors results in diminished tumor cell proliferation (7, 32–34). Transferrin receptors on the cell surface are upregulated in aggressively growing GBMs possibly to meet their high metabolic demand for iron (35). However, they may also be upregulated as a result of iron deprivation (9). Iron is directly taken up by cells through TfR1-mediated endocytosis. These receptors are highly expressed on the surface of GBM cells (36, 37), which makes them an accessible target for TfR-directed therapies. Gallium is a group-IIIa metal with known antineoplastic activity in certain malignancies, *in vivo* and *in vitro* (38–41), yet this metallodrug remains underexplored in brain tumors. Whelan et al. (1991) reported that growth-inhibitory effects of gallium on brain tumor cells *in vitro* closely correlated with cellular iron uptake (42), which is in line with the pathway proposed previously (43). The mechanism by which gallium exerts its cytotoxicity is by interfering with the cellular iron homeostasis. Ionic gallium, Ga^3+^, resembles ferric iron, Fe^3+^, in radius, which enables gallium to replace iron in bacterial and mammalian iron-transporters and Fe^3+^-containing enzymes (44). Gallium avidly binds to Tf, which facilitates the uptake of both iron and gallium by cells via TfR-mediated endocytosis. However, since the two cations differ chemically (45), Ga^3+^ cannot be reduced, and when incorporated, it inactivates Fe^3+^-dependent reduction and oxidation processes necessary for bacterial and mammalian cell proliferation (44). Subsequently, intracellular gallium directly interferes with cell proliferation by inhibition of ribonucleotide reductase, an iron-containing enzyme vital for DNA synthesis and repair (46), and triggers apoptosis through the mitochondrial pathway by activating proapoptotic BAX and activating caspase-3 (47, 48). Moreover, we recently demonstrated that GaM blocks mitochondrial complex I activity, significantly lowers cellular oxygen consumption rates of GBM cells *in vitro* and increases cellular ROS production. This cytotoxic inhibition of mitochondrial function was synergistically potentiated by metformin (49). Studies are ongoing to further probe the mechanisms of antineoplastic action of GaM in trGBM and other brain malignancies.

Our findings demonstrate that *oral* GaM effects a disruption of trGBM iron metabolism. Administration of GaM did not appear to affect expression levels of ferroportin protein in cancer cells, reinforcing its interference with iron uptake, rather than export, from cancer cells. It implies that whereas ferroportin is responsible for the export of iron out of cells (50), gallium may not require ferroportin to exit cancer cells. Preliminary *in vitro* studies by our group in a panel of pediatric brain tumor cell lines, including GBM, suggest that gallium may be cleared via lysosomes (51). Further investigation is needed to determine whether there is a role for this transporter in the cellular handling of GaM.

The resultant iron homeostasis caused a significant anti-proliferative effect, evidenced by an absence of invasive margins and a significantly lower proliferative index in the treatment group. The GaM-treated trGBM tumors in our study grew at a significantly slower mean weekly rate compared to untreated ones during their respective study periods. Moreover, the mean contrast-enhancing lesion volumes of GaM-treated tumors were significantly smaller than those of untreated intracranial tumors at matched timepoints. One rat in the GaM group also experienced complete resolution of the tumor. To our knowledge, this type and magnitude of response has not been observed previously in a preclinical intracranial model of GBM following administration of any conventional drug-based therapy. Instead, the survival outcomes using standard chemotherapy regimens in an intracranial xenograft model of GBM have been modest, at best (25, 26). Importantly, long-term treatment with oral GaM significantly extended the median disease-specific survival of animals to almost twice that of untreated controls. Diminished morbidity in both duration and severity relative to controls, normal liver and kidney function, as well as normal body condition in GaM-treated animals at the time of death further imply a quality-of-life benefit. This may translate to a substantial clinical and survival benefit in patients (52).

Notable cell distension was seen histologically in tumor tissues corresponding to central non-enhancing areas on MR images in GaM-treated animals. This suggests that the lesion expansion and the subsequent mass effect leading to death in the GaM group is likely due to treatment (53). Mount et al. also observed considerable tumor mass effect in mice bearing orthotopically engrafted patient-derived midline gliomas treated with anti-GD2 CAR T cell therapy (54). In that study, treatment-associated inflammation within the tumor site resulted in hydrocephalus, increased intracranial pressure, and transtentorial herniation in responding mice, ultimately resulting in death. Consequently, we propose that the increases in lesion volume and associated mass effect seen after appearance of these changes on post-contrast T1w MRI in GaM-treated rats in our study are likely due to edematous cells created by treatment effect rather than progressive tumor growth. We favor this as the most plausible explanation, since intracellular edema is a common feature following cytotoxic insult and radiation therapy (53, 55). It is also observed in necrotic tissues following traumatic brain injury (56). Clinically, it might be possible that by abating this edema with appropriate supportive therapy, such as steroids or anti-angiogenic agents which treat necrosis (57), the treatment window could be extended, thus allowing tumor regression to fully take effect. Additionally, in the clinical setting, the majority of patients with progressive or recurrent disease will have undergone surgery previously, and in the presence of a resection cavity, the cytotoxic edema caused by GaM might not be as consequential as in our animal model. Future studies will incorporate therapies to counteract the treatment-related mass effect.

Immunohistochemical evidence of cytotoxicity in GaM-treated xenograft tumors in the presence of intact vascular endothelial cells imply a sparing effect of normal brain structures. The observed morphological changes in the vasculature, such as prominent vessel pruning, loss of branching, and uniformity of vessel lumina, are suggestive of vessel normalization. Such a normalization is desirable clinically, as it is thought to alleviate hypoxia and promote the delivery and efficacy of conventional chemotherapeutics (22, 58). *In vitro* and *in vivo* studies show that in response to hypoxic stimulation, ANG2 expression, in the absence of VEGF, is associated with vessel regression in gliomas (59, 60). We also observed such ANG2 and VEGF expression patterns following GaM treatment. Targeting tumor angiogenesis by inhibiting VEGF is a popular strategy to remodel the tumor vasculature and bring about vessel normalization (61). Clinical and preclinical MRI studies demonstrate that anti-VEGF therapy with either cediranib or bevacizumab normalizes the brain tumor vasculature by decreasing vessel diameter and permeability (62, 63). Unfortunately, because the major vascular remodeling induced by anti-VEGF treatment leads to a more hypoxic tumor microenvironment, this vessel normalization typically is only of a temporary nature (64). Such is the case with bevacizumab, where treatment-induced hypoxia in the tumor microenvironment effects a metabolic shift in the tumor cells toward glycolysis, which, in turn, leads to a dramatic increase in parenchymal tumor cell infiltration into the normal brain (63, 65). Whereas we noted a decrease in VEGF expression on IHC in our study, we did not observe any concurrent brain invasion in GaM-treated xenograft tumors. Work from our collaborators demonstrated that GaM decreased the expression of pro-angiogenic cytokine IL-10 in a mouse model of cutaneous T-cell lymphoma, whereas angiostatic proteins CXCL10 and CXCL11 were significantly upregulated (66). While the expression levels of these cytokines remain to be examined in our *in vivo* trGBM xenograft model, it is plausible that GaM may exert its anti-angiogenic properties via more than one mechanism. Mass spectrometry-based proteomics studies utilizing saved tissue and plasma specimens are planned to elucidate the complex interplay of VEGF, ANG2 and hypoxia during GaM treatment.

Attempts at measuring serum VEGF levels as a diagnostic or survival marker in patients with high-grade brain tumors have yielded conflicting results (67), likely because GBMs modulate VEGF secretion locally (68, 69). Thus, serum VEGF levels may not be sufficiently elevated to properly reflect what is happening at the tissue level in response to antiangiogenic therapy. Perfusion MRI may correlate to tumor angiogenic markers, such as VEGF (70–72), rendering it a suitable non-invasive method to monitor vascular changes during treatment. It is an invaluable clinical tool for diagnosis of brain malignancies and treatment monitoring (58, 62, 63, 73–75). As such, it is part of the standard imaging protocol for brain tumor patients at many institutions, including ours. In this study, treatment with GaM resulted in an overall blunting of the MRI perfusion measure, ssCBV, compared to controls, although a spike was observed a week prior to the exacerbated tumor growth commencing at day 49. We propose that this was caused by hypoxia-driven angiogenesis in response to treatment-induced necrosis (22, 76, 77). This “angiogenic switch” is a well-documented survival mechanism of cancer cells and a driver of angiogenesis, which ultimately leads to tumor progression and invasion (77–79). A more measured intermittent or metronomic dosing system may be advisable for future studies to bring about a sustained curbing of tumor growth (80–82). Alternatively, GaM may require combination with other, synergistic agents, such as metformin, for a more potentiated anti-tumor effect (49).

Sex differences in perfusion measures with and without GaM treatment were evident. Our own observation of females having higher perfusion values than males is corroborated by others. Although the exact reason for these findings is unclear (83), this phenomenon may be due to sex differences in vascular response to insult (84). The influence of sex on GBM pathophysiology is complex and still not well understood. We have only just begun to probe the complexity of how sex influences iron targeted therapy in trGBM. In our studies both male and female rats were inoculated with the same tumor cells, as well as housed, treated and imaged under the same conditions. It could be argued that our observed difference in trGBM response to treatment may amount simply to individualized responses. Another possibility is that our female cohort was underpowered. Since we could not predict the magnitude of response to treatment in either sex, we applied the same power analysis for both male and female cohorts. For future studies, we will apply a separate power analysis for each sex to account for differences in incidence and magnitude of treatment effects. However, it is more plausible that inherent sex-dependent metabolic differences, at the cellular level and in the tumor microenvironment, are at play. Iron metabolism and iron biodistribution varies between the sexes, in part driven by differences in hepcidin, ferritin and ferroportin expression (85, 86). Further, there are sex differences in iron metabolism, particularly relating to binding of iron by transferrin and ferritin (87). How exactly these differences influence response to GaM is being investigated in ongoing studies.

Gallium maltolate has the distinct advantage over gallium salt formulations, such as gallium nitrate, which must always be administered intravenously, in that it may be ingested. Moreover, GaM has a high oral bioavailability and a greater therapeutic index that translates into greater antineoplastic efficacy and a potentially lower incidence of adverse side-effects than gallium nitrate (88). Our results confirm the safety profile of GaM. In our study, mean compliance with voluntary ingestion of GaM was high, which reassured us that all animals received a total to near-total of the calculated daily dose of GaM. Animal appetites decreased slightly for 24 - 48 hours following prolonged anesthetic events (MRI sessions), but this was adjusted for by slightly decreasing the amount of vehicle on those days. No adverse effects to body condition or changes in behavior were noted during the treatment period. The absence of blatant toxic effects was confirmed on necropsy and by histology. All brain structures surrounding treated lesions, including the vasculature, were judged to be normal and healthy in appearance. This further validates previous *in vitro* work by our group demonstrating that brain vascular endothelial cells are unaffected by gallium (9).

The work presented here has served as the springboard for a Phase I clinical trial (ClinicalTrials.gov ID: NCT04319276) at our institution which launched earlier this year. Dose regimens for GaM alone and in combination with standard therapy remain to be determined for optimal clinical impact. In ongoing work, we seek to evaluate GaM in combination with other therapeutics to translate them for clinical use, as well. Further efforts in our lab involve the development of diagnostic and prognostic advanced imaging biomarkers. Recently, we demonstrated that basal ganglia iron levels may be a useful biomarker in glioma prognosis (89). Advanced MR imaging techniques already have the potential to predict the influence of sex on GBM outcomes (16, 90). Finally, on PET/CT, ^68^Ga tracer uptake was found to correlate to brain tumor grade, with the ability to distinguish between low and high grade, as well as between grade III and IV, gliomas (91). Thus, the added potential exists for the development of imaging biomarkers specifically for the monitoring of GaM therapy in the clinical setting. Taken together with data gleaned from our Phase I clinical trial, our work will inform future truly personalized sex-specific treatment regimens.

In conclusion, the importance of iron in cancer biology and the high requirement for iron by GBM tumors make tumor iron homeostasis and iron-dependent proteins attractive targets for therapeutic intervention. Mounting evidence from our group suggests a unique role for iron-mimetic gallium compounds in trGBM therapy, since iron metabolism is ubiquitous to all cancer cells. Moreover, the GaM formulation shows unparalleled promise due to its high oral bioavailability and acceptable therapeutic index. As an orally administered therapeutic it further promises ready patient acceptance into their regular chemotherapy regimen. If the results in our trGBM rat model translate to a comparable patient experience, targeting iron metabolism as an adjunct to standard-of-care may transform clinical outcomes and effect a momentous shift in the treatment paradigm of trGBM.

## Data availability statement

The raw data supporting the conclusions of this article will be made available by the authors, without undue reservation.

## Ethics statement

The animal study was approved by the Medical College of Wisconsin Institutional Animal Care and Use Committee. The study was conducted in accordance with the local legislation and institutional requirements.

## Author contributions

MA-G: Conceptualization, Data curation, Formal analysis, Investigation, Methodology, Validation, Writing – original draft, Writing – review & editing, Project administration. RW: Data curation, Formal analysis, Methodology, Software, Writing – review & editing. HA: Data curation, Methodology, Writing – review & editing, Conceptualization, Formal analysis. JCob: Formal analysis, Writing – review & editing. MP: Writing – review & editing, Formal analysis. ND: Formal analysis, Methodology, Data curation, Writing – review & editing, Conceptualization, Resources. JCon: Conceptualization, Visualization, Writing – review & editing. CC: Conceptualization, Methodology, Resources, Supervision, Visualization, Writing – review & editing. KS: Conceptualization, Funding acquisition, Investigation, Methodology, Project administration, Resources, Software, Supervision, Visualization, Writing – review & editing.
