# Supplementary figures and images for: Potent in vivo efficacy of oral gallium maltolate in treatment-resistant glioblastoma
Source: Front Oncol. 2024 Jan 15;13:1278157. doi: 10.3389/fonc.2023.1278157 (PMC10822938; doi:10.3389/fonc.2023.1278157)

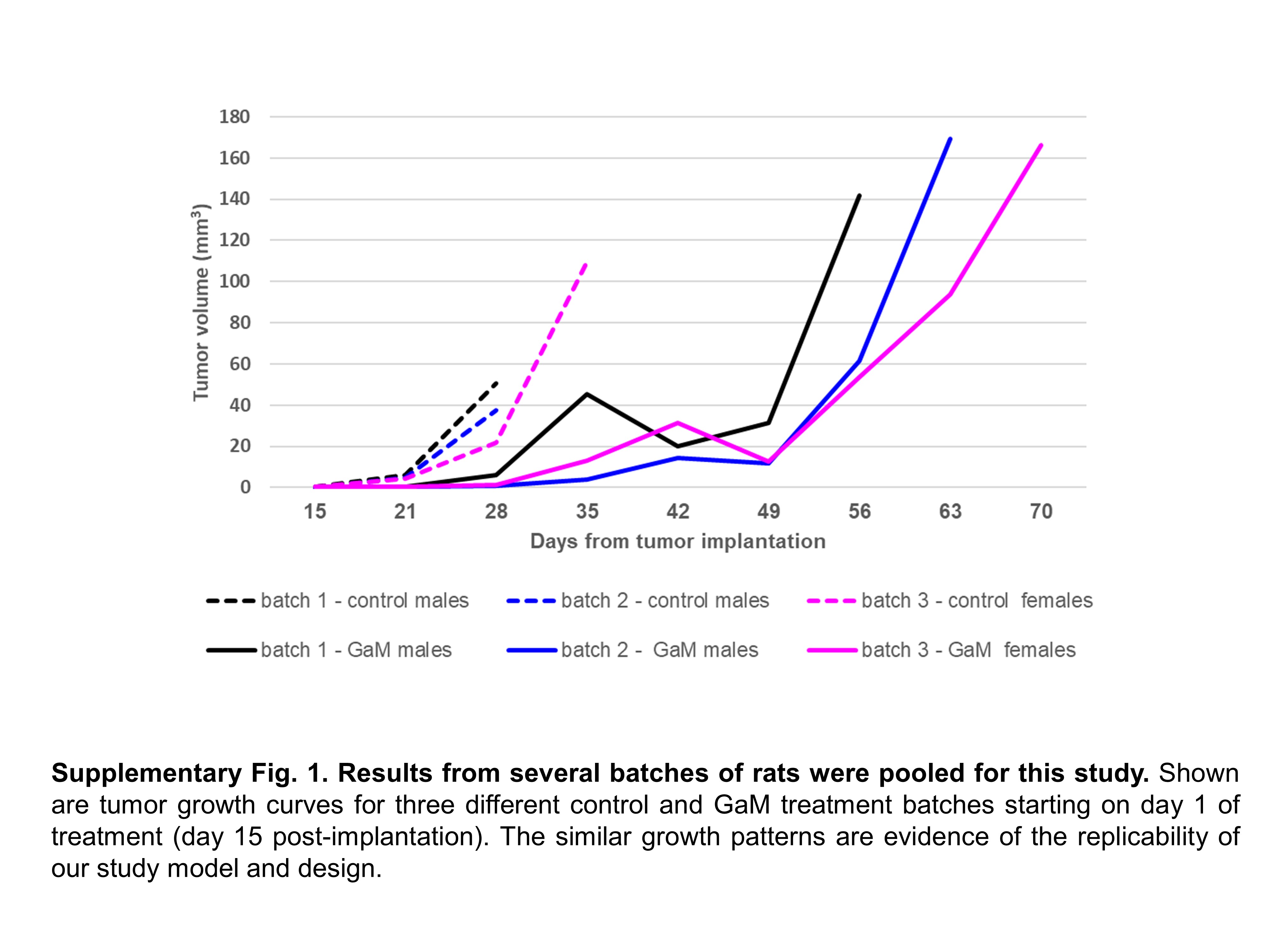

Supplement: Supplementary file 1 [file Image_1.jpeg]

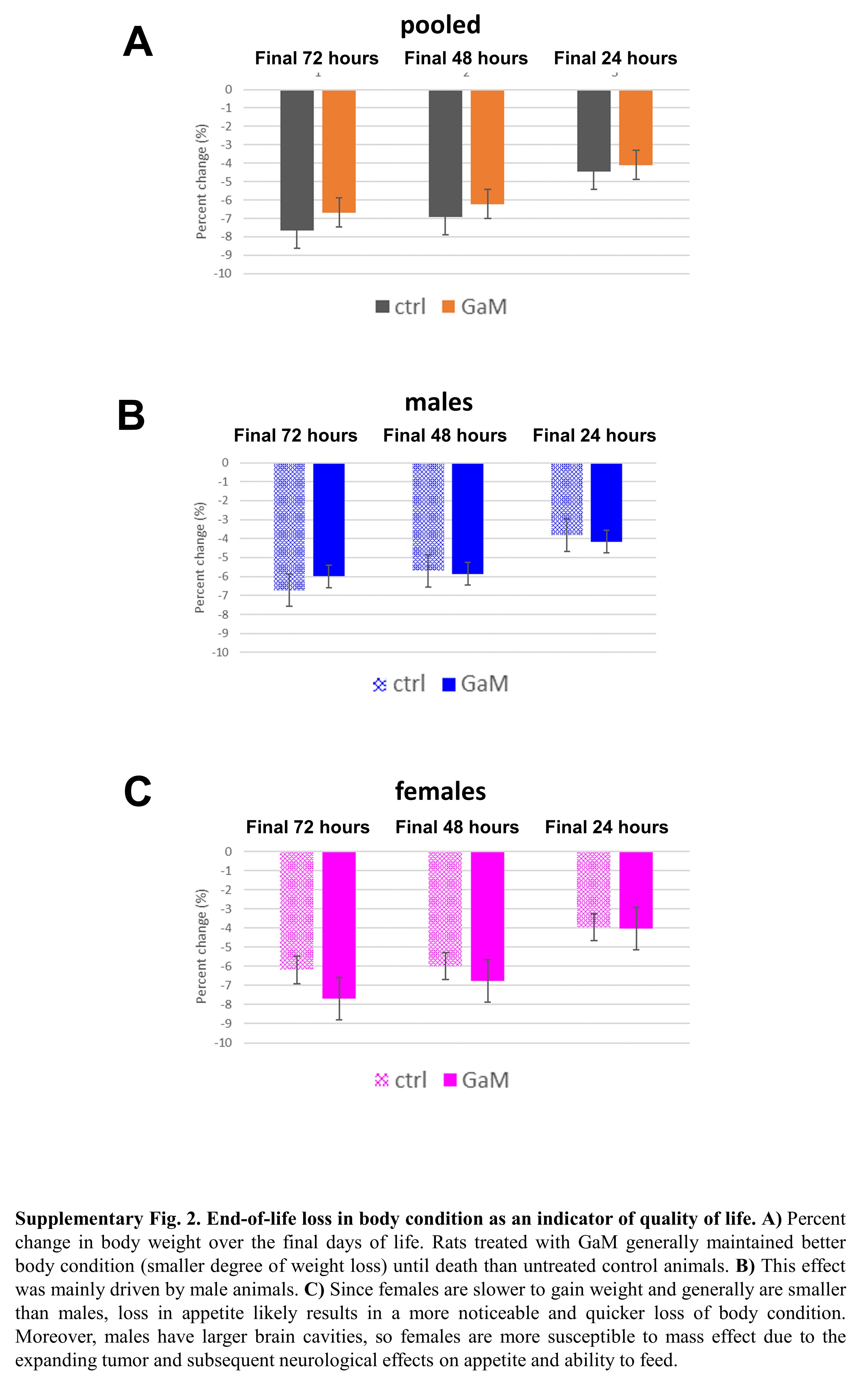

Supplement: Supplementary file 2 [file Image_2.jpeg]

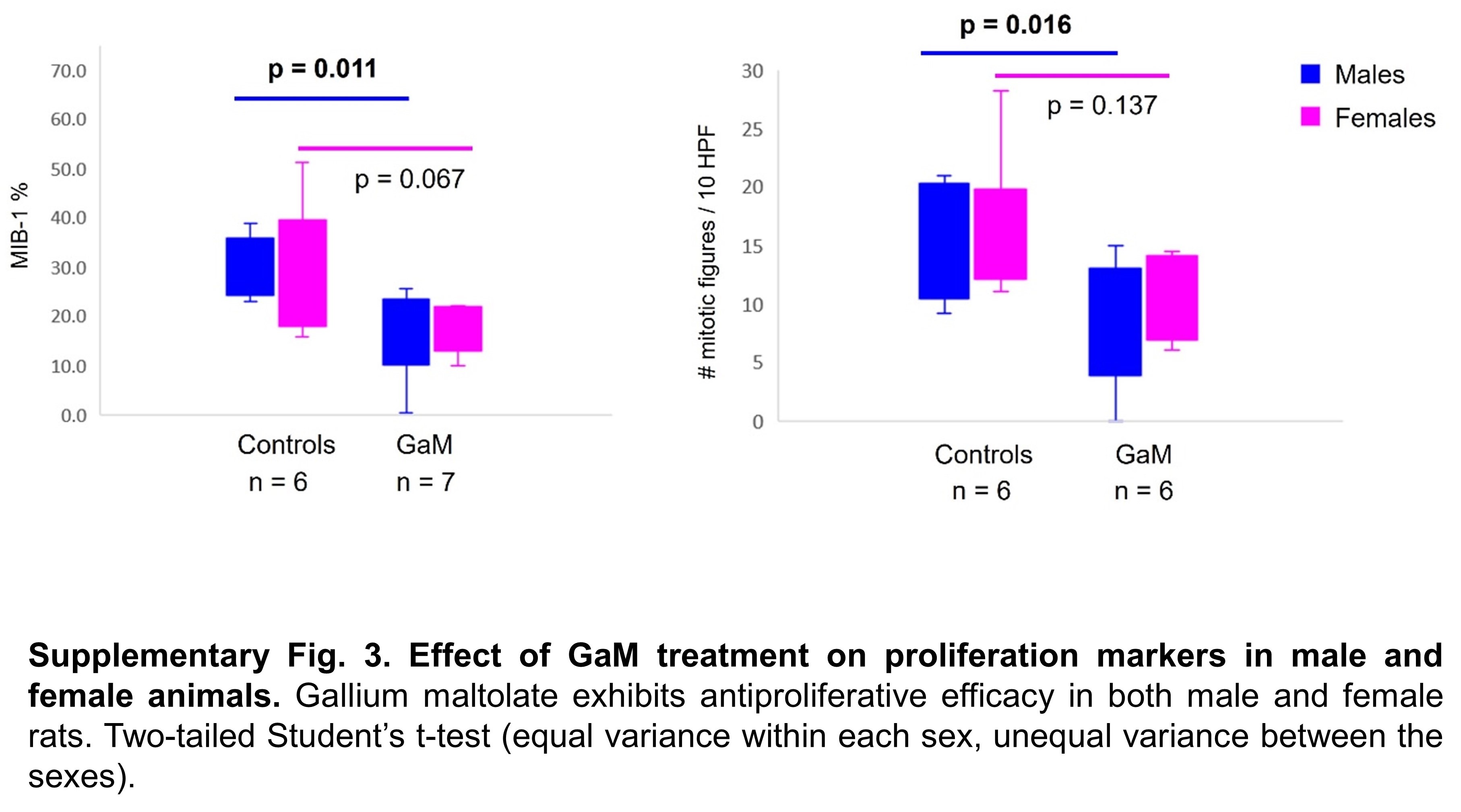

Supplement: Supplementary file 3 [file Image_3.jpeg]

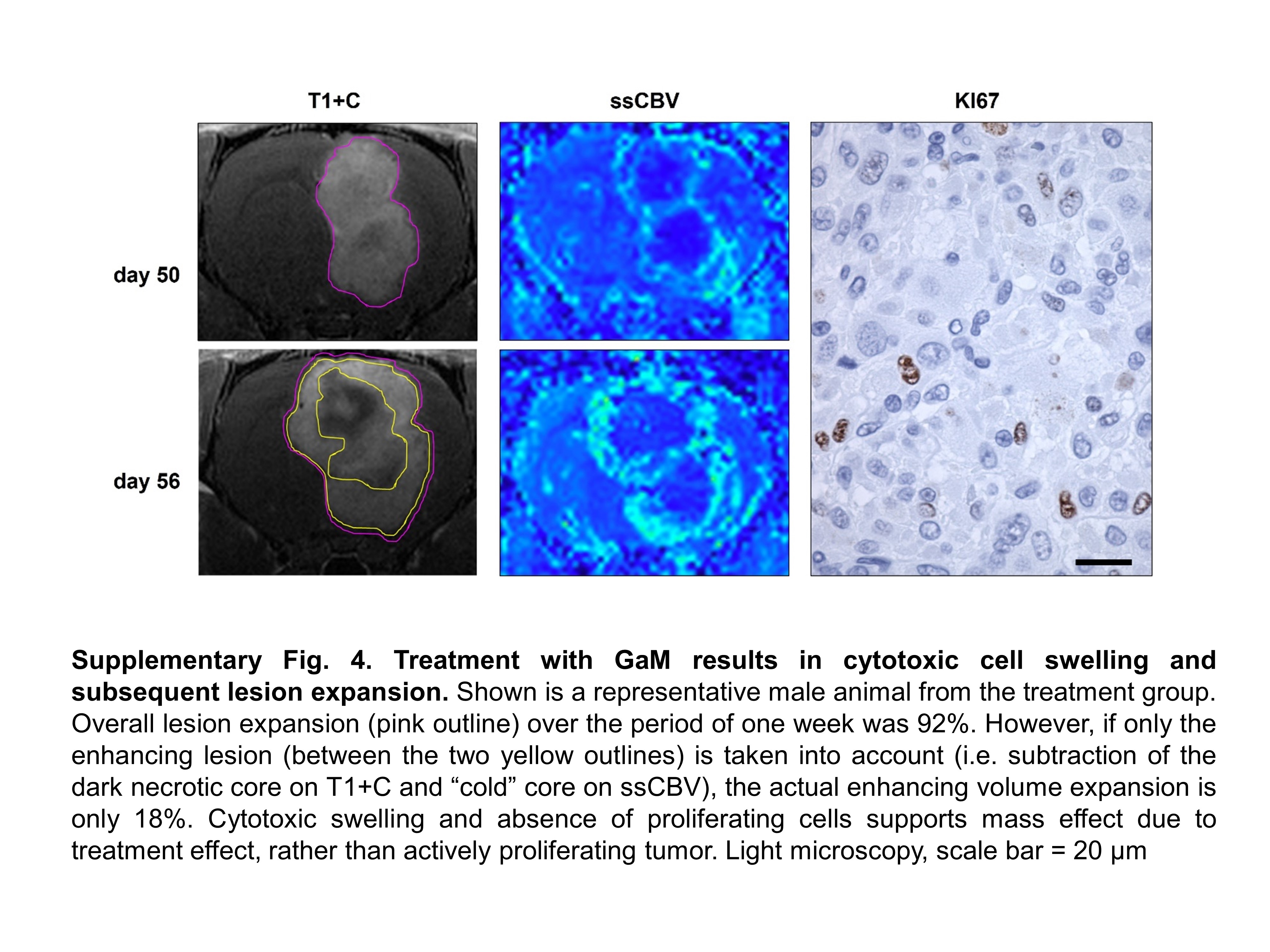

Supplement: Supplementary file 4 [file Image_4.jpeg]

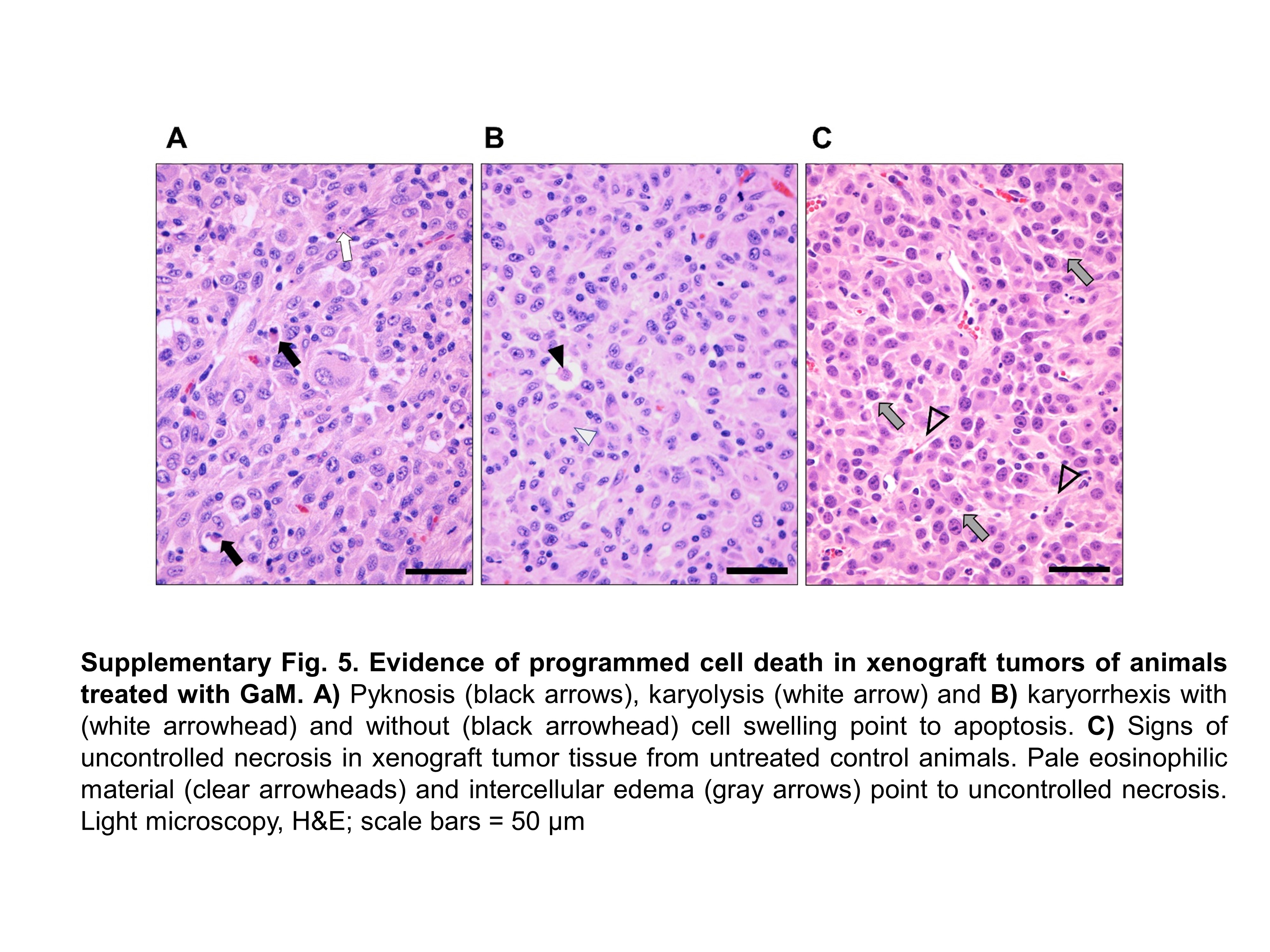

Supplement: Supplementary file 5 [file Image_5.jpeg]

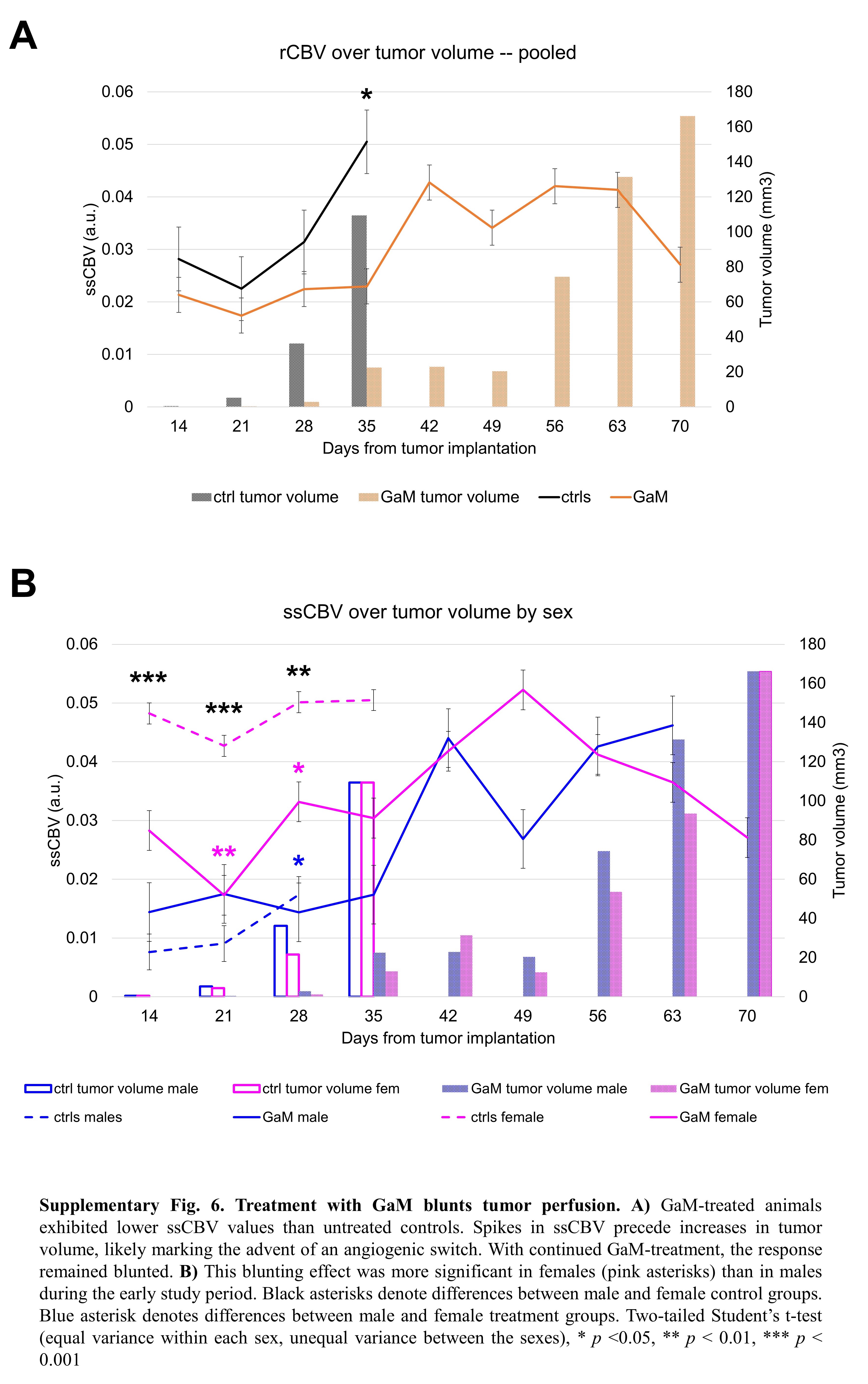

Supplement: Supplementary file 6 [file Image_6.jpeg]
